# Fe–S cluster assembly in the supergroup Excavata

**DOI:** 10.1007/s00775-018-1556-6

**Published:** 2018-04-05

**Authors:** Priscila Peña-Diaz, Julius Lukeš

**Affiliations:** 1 Institute of Parasitology, Biology Centre, Czech Academy of Sciences, České Budějovice (Budweis), Czech Republic; 2 Faculty of Sciences, University of South Bohemia, České Budějovice (Budweis), Czech Republic

**Keywords:** Fe–S cluster, Mitochondria, Excavata, Evolution

## Abstract

The majority of established model organisms belong to the supergroup Opisthokonta, which includes yeasts and animals. While enlightening, this focus has neglected protists,  organisms that represent the bulk of eukaryotic diversity and are often regarded as primitive eukaryotes. One of these is the “supergroup” Excavata, which comprises unicellular flagellates of diverse lifestyles and contains species of medical importance, such as *Trichomonas, Giardia, Naegleria, Trypanosoma* and *Leishmania*. Excavata exhibits a continuum in mitochondrial forms, ranging from classical aerobic, cristae-bearing mitochondria to mitochondria-related organelles, such as hydrogenosomes and mitosomes, to the extreme case of a complete absence of the organelle. All forms of mitochondria house a machinery for the assembly of Fe–S clusters, ancient cofactors required in various biochemical activities needed to sustain every extant cell. In this review, we survey what is known about the Fe–S cluster assembly in the supergroup Excavata. We aim to bring attention to the diversity found in this group, reflected in gene losses and gains that have shaped the Fe–S cluster biogenesis pathways.

## Introduction

The intimate relationship between eukaryotic cells and mitochondria, as endosymbiont-derived organelles, has taken billion of years to establish [1, 2]. It was initially accepted that the basis for the presence of mitochondria in virtually every eukaryotic cell had been the provision of energy from their oxidative phosphorylation machinery, yet several lines of evidence have proven otherwise [3]. The description of various types of mitochondria and mitochondria-related organelles (or MRO), many of which are found in a spectrum of unrelated protist clades, has brought into the spotlight an enormous organellar diversity, or what is rather a continuum, ranging from a minimalistic MRO of *Giardia* to the highly complex mitochondria of trypanosomes [4]. MROs have been found in eukaryotic supergroups Excavata, SAR (stramenopiles/alveolates/Rhizaria), photosynthetic algae, and Opisthokonta [1, 5]. Organelles of mitochondrial origin, of which MROs form a part, have been classified into five types [5]: (1) “classical mitochondrion” with a complete electron transport chain, which is capable of using oxygen as an electron acceptor, and produces metabolic energy from such machinery; (2 and 3) organelles that bear a functional electron transport chain, yet use other electron acceptors such as fumarate, and are capable of performing both substrate-level phosphorylation and may or may not produce H_2_; (4) double membrane-bound MROs called hydrogenosomes that are capable of ATP production in anaerobic or microaerophilic environments and excrete H_2_ as one of the end products of substrate-level phosphorylation in an organelle lacking electron transport chain [6]; finally, (5) mitosomes represent a type of MRO incapable of energy production, as they lack the components of an active electron chain and mostly have lost their genome [1, 5].

Remarkably, the Fe–S cluster assembly pathway is the only known common denominator of this conglomerate of organelles. Moreover, in one of the best-studied eukaryotes, the yeast *Saccharomyces cerevisiae*, this pathway seems to be the only truly essential component of its mitochondria [3, 7]. In this review, we revise what is known about the Fe–S cluster assembly pathways of the unicellular supergroup Excavata and attempt to compare its inventory with those described in other eukaryotes, but mostly in the Opisthokonta, the by far most studied supergroup that includes, not surprisingly, humans [8].

Fe–S clusters are ancient cofactors found in the whole spectrum of life. In Archaea, Bacteria and Eukaryota, cells have evolved different machineries for their Fe–S clusters assembly, namely the methanoarchaeal sulfur mobilization (SUF) machinery [9], the nitrogen fixation (NIF) pathway [10], the cysteine sulfinate desulfinate (CSD) system [11], the iron–sulfur cluster assembly (ISC) pathway [12, 13] and cytosolic iron–sulfur protein assembly (CIA) pathway [14–16]. The first one is present throughout bacteria and in the chloroplasts of photosynthesizing organisms [17], while the NIF system was initially discovered in the maturation of nitrogenase in N_2_-fixing bacteria [18]. The CSD system has been described in *Escherichia coli* as a partial functional homolog of the SUF system [19]. The ISC pathway is associated with eubacteria and in eukaryotes located within the mitochondria [3]. Finally, the CIA machinery is found in the cytosol of eukaryotes, where it assembles, with the assistance of mitochondrial ISC, Fe–S clusters eventually incorporated into proteins located in this cellular compartment [14, 20]. It is now widely accepted that the latter pathway was bequeathed from an endosymbiont to an ancestral eukaryote, while the plastid-bearing eukaryotes inherited their SUF machinery from an early cyanobacterium [17, 21, 22]. On the other hand, a lateral gene transfer has been proposed to be behind the emergence of the SUF pathway in some unicellular eukaryotes that either retain highly diverged MROs, such as *Blastocystis*, *Stygiella incarcerata* and *Pygsuia biforma*, or lost the entire organelle, along with the ISC pathway, like in the case of *Monocercomonoides* [23]. In any case, the Fe–S cluster assembly represents a hallmark that is always taken into account when analyzing the evolution of divergent mitochondria-derived organelles [12, 24–28].

## Fe–S cluster machineries: a brief overview

### Bacterial systems

The Fe–S cluster assembly machineries in bacteria can be subdivided into four systems mentioned above, their distribution being species specific [18, 29, 30]. As an example, *E. coli* with the best-studied bacterial Fe–S assemblies exhibits the ISC, the SUF and the CSD systems [18, 19]. These distinct systems share the main activities for the formation of a [2Fe–2S] cluster, which may be divided into three main stages: (1) Pyridoxal 5′-phosphate-dependent cysteine (Cys) desulfuration with concomitant production of l-alanine, carried out by a cysteine desulfurase (CD). The ISC, SUF, CSD and NIF systems each bear a functional homolog of this enzyme which, however, displays significant differences addressed below. (2) Assembly of an Fe–S cluster on a scaffold protein; a Cys in the scaffold protein binds sulfur liberated from the previous reaction, which physically interacts with the CD for the Fe–S assembly to occur. (3) Transfer of the Fe–S cluster from the CD–scaffold complex onto a delivery protein, which will subsequently interact with the recipient proteins and install the cluster.

#### The ISC system

The Fe–S cluster assembly by the ISC system involves the activity of at least five proteins. A cysteine desulfurase (IscS) provides the sulfur, while the formation of the cluster takes place on the scaffold protein named IscU. Ferredoxin (Fdx) likely acts as an electron donor in this reaction [31, 32]. Subsequently, IscU requires the assistance of the chaperones HscA (heat shock cognate 66 kDa or Hsc66) and HscB (heat shock cognate 20 kDa or Hsc20). HscA is a member of the Hsp70/DnaK chaperone family and exhibits an intrinsic ATPase activity, while HscB belongs to the Hsc20/DnaJ J-type co-chaperone family [33]. The binding of the chaperones, which is mediated by an LPPVK motif found in IscU, occurs in an orderly fashion to release the nascent Fe–S cluster onto the delivery system in an ATP-dependent manner [34–37]. In this scenario, HscA binds IscU, enhanced by the presence of HscB, which increases the ATPase activity of the former chaperone by approximately 400-fold [35, 38]. The binding model formulated based on the studies of the IscU mutants proposes that the chaperone complex stabilizes IscU for the release of the cluster to the receptor protein [39]. IscU has also been observed to interact with both [2Fe–2S] and [4Fe–4S] clusters [40]. This was further analyzed upon the binding of the scaffold protein to Fdx, which has been proposed to mediate, at least partially and in vitro, the reductive coupling of [2Fe–2S] clusters into [4Fe–4S] clusters [41]. Once the Fe–S cluster has been assembled, it is transferred to the delivery system for further insertion into apoproteins, a role carried out by the dedicated protein IscA. The bacterial frataxin, named CyaY, plays an inhibitory role in the biogenesis of the Fe–S cluster assembly by binding to IscS in an iron-sensing regulatory role [42].

#### The SUF system

The SUF system for the Fe–S cluster assembly is induced by iron-depleted and oxidative stress conditions and mainly involves the activity of two sub-complexes [9]. Proteins SufE and SufS form the heterodimer SufSE, while a second sub-complex termed SufBCD is composed of SufB, SufC and SufD [43, 44]. SufS, which represents the CD and therefore is an ortholog of IscS, collaborates with SufE, which increases the desulfurase activity of the CD [43, 45, 46]. SufSE transfers the sulfur produced by the CD reaction, following the interaction with the scaffold complex SufBCD, which further enhances the desulfurase activity [47]. SufB has been defined as a scaffold, capable of interacting with SufD and the soluble ATPase SufC [45, 48, 49]. Moreover, this complex uses FADH_2_ as a redox cofactor [48]. SufD shares substantial sequence similarity with SufB and has been hypothesized to confer iron to the reaction [50, 51]. The stoichiometry plays an important role in the structural dynamics of the SufBCD complex, with its various oligomeric forms being capable of transferring in vitro the cluster to the receptor proteins [49, 50]. For in vitro maturation of Fdx, the SufBC_2_D, SufB_2_C_2_ and SufC_2_D_2_ sub-complexes interact with SufA, which has been proposed to act as a transfer protein for the nascent Fe–S cluster [52]. However, it has also been established in vitro that the SufBCD complex is capable of transferring clusters directly to apoproteins without the assistance of SufA [49]. Once the Fe–S cluster is assembled, it is ready to be targeted to the transfer proteins. The SUF system exhibits an A-Type carrier protein termed SufA. [49]. While SufA belongs to the *suf* operon and IscA to the *isc* operon, another transfer or carrier protein called ErpA is independent of the Fe–S cluster assembly operons [53]. A-type carrier proteins were initially believed to act as alternative scaffolds [31], but several lines of evidence argued against this premise. It was observed that deletions of the type-A carrier produced no phenotypes, probably a reflection of the fact that the nascent cluster can be transferred directly from the scaffold protein [54]. Moreover, the binding capacity of type-A carriers did not allow reversible transfer of the Fe–S cluster from the scaffold, and their binding to CD was not as efficient as that of IscU [55]. For these reasons, two possible roles in Fe–S assembly were considered: they could either act as iron donors [56] or assist the process of transfer to the apoproteins once the Fe–S cluster was assembled [49].

Another protein belonging to the SUF system is the Zn-dependent SufU [57], which is absent in the Gram-negative bacteria such as *E. coli*. SufU, a homolog of IscU, is able to complement SufE, as it happens in the case of *Bacillus subtillis, Enterococcus faecalis, Thermatoga maritima* and various mycobacteria [58–61].

#### The NIF system

The NIF system, initially described in nitrogen-fixing bacterium *Azotobacter vinelandii*, is a dedicated machinery for the maturation of nitrogenase. Later on, it has also been found co-existing with one or more of the other three Fe–S assembly systems in ε-proteobacteria, represented by *Helicobacter pylori* [62], as well as in the Gram-negative γ-proteobacteria *Dickeya dadantii* [63, 64] and *Klebsiella pneumoniae* [65]. The NIF system comprises a CD termed NifS and a scaffold protein NifU. NifU is a functional homolog of the scaffold protein IscU, yet it exhibits some remarkable differences. On its N-terminus it bears cysteines intended for [2Fe–2S] cluster binding, while other cysteines found in its middle and its C-terminus seem to bind Fe–S clusters in a non-transient manner [66]. NifB is an S-adenosylmethionine (SAM)-dependent enzyme involved in the formation of an Fe–S cluster precursor of an iron–molybdenum cofactor (FeMo-co) required for the reconstitution of active nitrogenase [67]. Other proteins involved in the NIF system are IscA^nif^, which likely functions as a scaffold for target apoproteins [32], and a *O*-acetylserine synthase denoted as CysE1, whose activity has been proposed to increment the cysteine pool for the Fe–S assembly of nitrogenase [68].

#### The CSD system

The CD of the CSD system is encoded by the *csdA* gene. It differs from SufA by substrate specificity, although both proteins share substantial sequence similarity. CsdA is capable of transferring sulfur from l-selenocysteine, l-selenocystine, l-cysteine, l-cystine and cysteine sulfinate [69]. CsdE, a homolog of SufE, catalyzes the release of Se, SO_2_ and S from l-selenocysteine (the most preferred substrate), l-cysteine sulfinate and l-cysteine (the least preferred substrate) [70, 71]. Regardless of the CD activity observed in vitro for each pure protein, labeling assays confirmed that CsdA and CsdE may enhance each other’s activity twofold [11, 72]. In *E. coli,* the CsdAE complex has been observed in unison with the SufSE and SufBCD sub-complexes. Under specific nutritional deficiency stress, the CsdAE complex may also recruit CsdL, an E1-like protein found in ubiquitin-like systems [73].

### Eukaryotic systems

Eukaryotic cells exhibit the ISC system in the mitochondria and the CIA pathway in the cytosol. The photosynthetic eukaryotes also carry the SUF system in their plastids [74]. Moreover, the SUF system has been retained even in the non-photosynthetic apicoplast of the apicomplexan parasites [74, 75], as well as in the cytosol of some parasitic and commensal amitochondriate protists [23]. Finally, the NIF system is rarely present in the mitochondria and cytosol (e.g., in *Entamoeba histolytica* and *Mastigamoeba balamuthi*) [76, 77], while parts of the CSD system were found scattered in some parasitic protozoan genomes [78].

#### The mitochondrial ISC system

The best-studied mitochondrial ISC systems are those of yeast, plants and mammalian cells. In this section, we will base our description on the human model and therefore use the nomenclature for the human ISC Fe–S cluster assembly, unless indicated otherwise. The ISC system from bacteria and eukaryotes exhibits the same general functional traits, such as the sulfur acquisition from a donor cysteine and the assembly of a nascent Fe–S cluster on a scaffold protein, with the subsequent transfer to carrier proteins, which will in turn deliver the cluster to apoproteins. However, there are some important differences between the prokaryotic and eukaryotic ISCs. One of them is the ISD11 protein, exclusive for eukaryotes [79]. ISD11 is a small accessory protein that acts in concert with NFS1 [80–83], the scaffold protein ISU2 and the acyl carrier protein ACP [84]. Within the NFS1–ISD11–ACP tripartite complex, ISD11 forms a dimeric association at its core, with ACP occupying a hydrophobic pocket in each ISD11 monomer that stabilizes the complex [85, 86]. The complex also binds to the scaffold protein ISCU2 and to ferredoxin (FDX2) [87], which acts as an electron donor aided by a ferredoxin reductase (FDXR) [88–90]. ISCU2 is the product of alternative splicing of *ISCU* which also gives origin to ISCU1, also suggested to play a role in the repair of the [4Fe–4S] clusters in the cytosol [91]. In humans, the binding is facilitated by the interaction with a chaperone/co-chaperone complex formed by HSPA9 (also called GRP75 or PBP74) and HSC20 (or HSCB) [92, 93], while in yeast this role is performed by mitochondrial Hsp70 protein, ATP-dependent Ssq1 and co-chaperone Jac1 [94, 95]. Frataxin (FXN) has been proposed to donate Fe^2+^ for the reaction and/or act as an allosteric regulator of NFS1, stimulating its activity [96–98]. Moreover, FXN has also been suggested to bind to the chaperone–ISCU2 complex [88]. The binding of ISCU2 to the L(I)YR motif seems essential for the transfer of the cluster to a specific recipient apoprotein [95]. Studies using succinate dehydrogenase complex (SDH) subunits in mammalian cells demonstrate that the clusters are transferred directly from the chaperone complex to the subunits of SDH, achieving their maturation in this step; a similar result was observed for respiratory complexes I and III [95]. Also a monothiol glutaredoxin, GLRX5, has been observed to interact with HSC20, through binding to the same group of cysteines that the chaperone uses to bind SDH [95]. Hence, GLRX5 must be released from the chaperone complex for it to bind to the recipient respiratory complex unit. Consequently, it was proposed that GLRX5 acts as an alternative scaffold for delivery of the Fe–S clusters to apoproteins [95].

An alternative model for the transfer of the cluster from the NFS1–ISD11–ACP–ISC2–FXN complex involves an interaction with GLRX5. This model implies that GLRX5 requires a chaperone-assisted transfer of the Fe–S cluster from the scaffold complex, which for most apoproteins cannot be bypassed [90]. Mechanistic data for this model have been obtained from the NMR spectra where the NFS1–ISD11–ISCU complex, aided by the chaperone complex is capable of transferring Fe–S clusters, although these experiments were performed in the absence of GLRX5 [99]. Another piece of evidence to support this model comes from in vitro experiments where GRX5 is capable of interaction with transfer proteins ISCA1 and ISC2, and also mobilizes their [2Fe–2S] clusters [100]. Co-immunoprecipitations of GLRX5 in mice N2a and HeLa cells also detected an interaction with carrier proteins ISCU2 and IBA57 [89]. Moreover, deletion of *GRLX5* in humans is known to cause severe microcytic-hypochromic anemia, related to Fe–S cluster biogenesis defects [101]. Regardless, the model involving GLRX5 also correlates with the formation and transfer of the [4Fe–4S] clusters (discussed in detail below), as the ISD11–ISC2–HSC20–HSC9 complex may only answer for the assembly of [2Fe–2S] clusters into the respiratory complexes I through III [90].

**Fig. 1:**
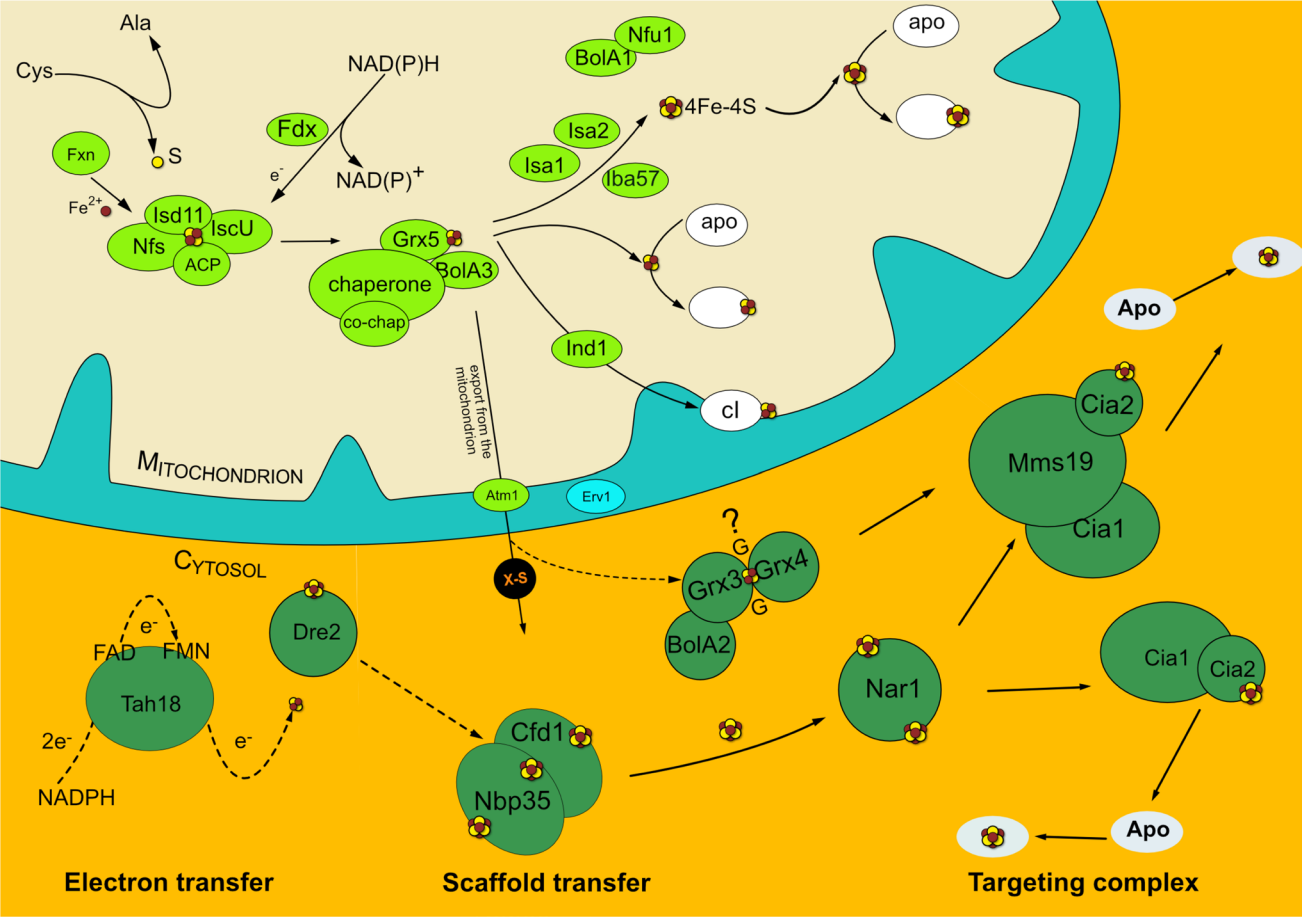
Schematic representation of the ISC and CIA pathways in eukaryotic cells. The generic representation displays ISC components (in light green) and CIA components (in dark green). Recipient proteins are depicted in white for ISC and blue for CIA. The CIA components reflect the complexes described for the mammalian cell model

The LYR-based transfer model described in human cells differs from the one proposed and demonstrated for yeast mitochondria [102]. We understand these two models are complementary, but we intentionally describe them separately, as they rely on different experimental setups. In yeast mitochondria, the chaperone Ssq1 binds to monothiol glutaredoxin Grx5 prior to binding to the scaffold protein Isu1, making use of two different binding sites for each protein [102]. The binding of Ssq1 to Grx5 is ATP independent, which entails that the ATP-dependent stage of Ssq1 activity takes place in the reaction upstream, while binding to the co-chaperone Jac1 (Fig. 1). Jac1 is then released from Ssq1 prior to the binding to Grx5, a step that precedes the binding to Isu1. The result is an efficient transfer of the newly formed cluster from Isu1 to Grx5 [102]. The model also implies the release of Grx5 bound to the cluster from the Ssq1–Isc1 complex, with the concerted activity of exchange nucleotide factor Mge1 [102]. The integration of Mge1 into this model comes from in vitro experiments, in which Mge1 is capable of releasing nucleotides bound to Ssq1 when the chaperone is in contact with Isu1. Therefore, the release of the chaperone from the scaffold (though these experiments where made, in the absence of Grx5 and in presence of Jac1), allows the continuation of a new cycle of assembly [103]. Mutations in *Grx5* in *S. cerevisiae* trigger a severe phenotype of iron accumulation and subsequent defects in the Fe–S cluster-bearing proteins [104]. The interaction of Grx5 with the Isa1 and Isa2 proteins has also been documented in the fission yeast *Schizosaccharomyces pombe* [105]. A yeast two-hybrid system provided additional evidence for the interaction between Grx5 and Isa1 in *S. cerevisiae* [106].

ISCA1 and ISCA2, along with IBA57, form the so-called ISA complex that has been proposed to act as a scaffold involved in the assembly of [4Fe–4S] clusters into a range of mitochondrial proteins, including lipoic acid synthase [89, 107]. Depletion of ISCA1 and ISCA2 in HeLa cells resulted in defects in the mitochondrial cristae formation, ultrastructure alterations and acidification of the growth media, which associates these proteins with the maturation of respiratory complexes and hence metabolic defects [107]. However, in mouse cell lines, silencing of these proteins did not cause any morphological alterations, yet respiration measurements corroborated the metabolic defects [89]. Silencing of IBA57 failed to exert an effect on the activity of the Fe–S cluster-carrying mitochondrial aconitase, yet it decreased in the activity of SDH. Similarly, the depletion of ISCA1, ISCA2 and IBA57 significantly lowered the activities of pyruvate dehydrogenase, 2-oxoglutarate dehydrogenase, mitochondrial aconitase, the Rieske protein and other Fe–S cluster-containing mitochondrial proteins. The phenotype could be reversed by RNAi-resistant ectopic expression of the two transfer proteins, proving their involvement in the phenotype [107]. However, [2Fe–2S] ferrochelatase was unaffected by silencing of ISCU1 in mouse, and of ISCU1, ISCU2 and IBA57 in human cells, corroborating their role only in late-acting stages of the Fe–S cluster assembly [89, 107]. Interestingly, when ISCU2 alone was silenced, increased amount of ISCU1 was observed in HeLa cells and vice versa, which led to the suggestion that these proteins may have redundant functions [107]. This is in partial agreement with human cases of leukodystrophy and hyperglycemia associated with defects in ISCA2 accompanied by a reduction in [4Fe–4S] clusters in respiratory complexes II and IV [108]. Alternatively, overexpression of ISC2 in an ISCU1 knock-down background in mouse cells showed that ISCA1, but not ISCA2, was essential for the [4Fe–4S] cluster assembly, since ISCU2 could not rescue the [4Fe–4S] defect caused by silencing of ISCU1 [89]. Regardless, the two scaffold proteins in mouse and human interact with each other in vitro [89, 109], although only ISCU2 was able to bind IBA57 [89].

The counterparts of human ISCU1 and ISCU2 in yeast are Isa1 and Isa2, which also function as scaffold proteins [110–112]. Experiments replacing Isu1 with bacterial A-type proteins in an *Isu1* mutant background corroborated that this protein, and not Isu2, is the functional ortholog of the bacterial counterpart [111]. Notably, Isu1 and Isu2 interact unlike their counterparts in humans [111]. Silencing of Isu1, Isu2 and Iba57 disrupts the activities of mitochondrial aconitase, biotin synthase and lipoic acid synthase, just like in human cells [110, 111, 113]. Regardless of whether Isu1, Isu2 and Iba57 are indeed directly involved in the [2Fe–2S] cluster assembly in both humans and yeast [100, 109, 114], there is a consensus that they all are late-acting components in the Fe–S assembly, directly involved in [4Fe–4S] cluster formation [115].

Another protein related to the Fe–S cluster assembly and mitochondrial respiratory complexes is the P-loop ATPase IND1. Silencing of IND1 caused a severe decrease of the Fe–S cluster-carrying complex I subunits, namely NDUFS1, NDUFV1, NDUFS3 and NDUFA13, leading to a defect in the assembly of the peripheral arm of the complex [116]. Depletion of Ind1 in the yeast *Yarrowia lipolytica* also causes a defect in complex I assembly [117].

The transfer of the Fe–S clusters to apoproteins involves the mobilization of the recently formed cluster to transfer proteins or assembly factors (called carrier proteins in bacteria), which will subsequently pass on the cluster to the recipient protein. In bacteria, this process has been defined to work in a “one-way” mode, i.e., is irreversible [19, 49]. The transfer protein NFU1 (or Nfu in yeast) is known to form homodimers and functions in mitochondria, cytosol and plastids [118–122], although in vitro experiments of the bacterial protein in *A. vinelandii* has been recorded forming heteromers [123]. The exact role of NFU1 in humans remains unclear, although defects in *NFU1* have been related to multiple mitochondria dysfunction syndrome [124–126]. In this syndrome, the lack of lipoic acid, due to the defective activity of lipoic synthase, causes the subsequent impairment in lipoid acid-dependent enzymes, such as PDH and KDH [126]. In vitro assays have determined that the protein interacts with ISCA1 [89] and BOL3 in humans [127]. Similar assays performed with yeast Nfu detected associations with Isa1 and Isa2, as well as with the [4Fe–4S] cluster proteins, Aco1, Aco2, lipoic synthase, biotin synthase and homoaconitase (Lys4) [120]. Yeast cells defective in Nfu displayed a relatively mild respiration impairment [120]. Yeast triple mutant *bola1*–*bola3*–*nfu* exhibited an enhanced phenotype unlike any of the individual mutants. Complementation experiments using human NFU1 or BOLA1 partially recovered the phenotype of the mutant, but not BOLA3, which indicates that a concerted activity of NFU1 and BOLA3 is necessary to recover the phenotype [120]. Moreover, the overexpression of Nfu in a *bola3* mutant recovered the respiratory defect phenotype, which indicates overlapping functions [120].

The obvious connection between Nfu and BolA assembly factors places them in similar, yet non-overlapping roles. BolA is a recently described protein of bacterial origin with three homologs in humans [128]. Yeast BolA1 and BolA3 localize to the mitochondria, while BolA2 is present in the cytosol [127]. BolA1 and BolA3 were shown to physically interact in vitro with human proteins NFU1 and GLRX5, respectively, providing further evidence that these proteins fulfill different functions. Indeed, *bola1 and bola3* mutants exhibit no growth phenotype, but the double *bola1*–*bola3* mutant displays a mild respiratory defect associated with decreased activities of enzymes related to the product of lipoic acid synthase, all of which require 4Fe–4S clusters [127]. Previous findings in yeast regarding BolA2 indicate that this cytosolic protein interacts with Grx3/4 as a chaperone [129].

A very important finding that defined the extent of the mitochondrial Fe–S assembly machinery was that of Atm1. A mutant strain for a mitochondrial ABC transporter in yeast exhibited iron accumulation in mitochondria, respiration defects and increased concentrations of glutathione [130]. Labeled iron assays in a mutant for Atm1 indicated that this protein did not affect the assembly of Fe–S clusters in mitochondria, yet it decreased the levels of the Fe–S cluster protein Leu1 in cytosol [131]. The transporter is located in the inner mitochondrial membrane, with its ABC domains facing the mitochondrial matrix, which implies an export activity mode [132]. This transporter likely transfers a sulfur derivative compound across the mitochondrial membrane, which is essential in cytosol.

#### The CIA pathway

Impairment of the mitochondrial Fe–S cluster assembly affects maturation of the Fe–S proteins in the cytosol of yeast and humans [133]. Indeed, the mitochondrial machinery is involved in the formation of a glutathione-based compound that is translocated to the cytosol to be integrated into the cytosolic Fe–S cluster assembly through the ABC transporter Atm1 (Fig. 1) [131, 134, 135]. The maturation of the Fe–S cluster proteins in the cytosol is performed by a different machinery from that of the ISC system that depends on substrates that must be exported out of the mitochondrion.

The cytosolic and nuclear de novo formation of [4Fe–4S] Fe–S clusters requires the heterodimeric association of proteins Cfd1 and Nbp35, which share significant sequence similarity [136–138]. The latter also shares homology with its bacterial and archaeal counterpart ApbC, both being classified as P-loop-containing nucleoside triphosphate hydrolases [139]. The ApbC/Nbp35 proteins were the first Fe–S cluster assembly proteins described in the Archaea [139]. Nbp35 exhibits an N-terminal Fdx-like domain, an ATP-binding motif and C-terminal cysteine residues [139]. The formation of the stable heterodimer depends on a shared [4Fe–4S] C-terminal cluster, labeled as bridging cluster, while both Nbp35 and Cfd1 each bear another [4Fe–4S] cluster in their N-termini [137, 140] (Fig. 1). Of importance is the fact that the N-terminal Fe–S cluster of Nbp35 is oxygen stable, unlike the bridging cluster [138, 140, 141]. Consequently, the N-terminal truncations render the protein unable to bear a cluster [137]. The Cfd1–Nbp35 conformation results in a heterotetrameric association capable of functioning as a scaffold for the assembly of an Fe–S cluster [140]. Remarkably, Cfd1 and Nbp35 are not targets of the de novo assembly factors of the CIA pathway, despite the fact that they themselves carry clusters. Their assembly and insertion depend on the early steps of the pathway [138]. Importantly, the Cfd1–Nbp35 heterotetramer formation does not take place in plants and certain anaerobic protists due to the absence of Cfd1 from their genomes [142, 143]. In humans, ablation of NBP35 causes an impairment in the iron-regulatory protein IRP1, which in turn affects the levels of ferritin and increases the levels of transferrin uptake by a concomitant increase of transferrin receptor levels in the cell. This effect on iron homeostasis involves the CIA machinery in a regulation role in mammals that is not observed in yeast [141].

The answer to which factors feed the stable Fe–S clusters to the scaffold protein Nbp35 came about with the characterization of Dre2 in yeast [144]. The deletion of Dre2 was lethal when combined with those of the mitochondrial carrier proteins Mrs3 and Mrs4 under iron-depleted conditions. The rationale behind this experiment was to identify protein(s) that provide a compound(s) for the cytosolic Fe–S cluster assembly, and resemble the phenotypes of CIA protein mutants known at the time [145]. Interestingly, although described as a cytosolic protein, low amount of Dre2 was also found either in the mitochondria and/or bound to the cytosolic side of the outer mitochondrial membrane [145, 146]. Dre2 is the yeast counterpart of anamorsin (or CIAPIN1, cytokine-induced apoptosis inducer 1) in humans, as proven by a complementation assay in yeast with the human gene [144, 145, 147]. The protein exhibits an N-terminal SAM methyltransferase-like domain, and on its C-terminus two motifs named I and II, which display cysteines required to bind the clusters [148]. In vitro experiments with overexpressed protein show that maturation of Dre2, which bears a [2Fe–2S] and a [4Fe–4S] cluster, is independent of the CIA factors, but requires the presence of Nfs1, positioning it functionally upstream of the Cfd1–Nbp35 scaffold step [144, 148]. Further analyses revealed that Dre2 interacts and functions in association with a diflavin reductase Tah18, which transfers electrons from NADPH_2_ to Dre2 (Fig. 1) [144]. At the same time, the human homolog of Tah18, NDOR, rescues the phenotype of *tah18* yeast mutants and cooperates with Dre2, which further strengthens the relationship between Dre2 and Tah18 [144]. However, in this model Dre2 is incapable of transferring clusters to apoproteins, but seems indirectly involved, together with Tah18, in the transfer of electrons for the formation of the [4Fe–4S] N-terminal cluster of Nbp35, yet has no effect on Cfd1 [144]. In human cells, CIAPIN1 has been found to interact with GLRX3, a cytosolic monothiol glutaredoxin [129, 147, 149] described below. Indeed, in vitro assays captured the transfer of a [2Fe–2S] cluster from the N-terminus of GLRX3 to the N-terminus of CIAPIN1. This result led to propose that the glutaredoxin feeds the [2Fe–S] cluster to CIAPIN1 [149]. However, this observation is in disagreement with the previously mentioned results from yeast, where the C-terminus of Dre2 is key for cluster binding [144]. Another piece of evidence postulates that the maturation of the [2Fe–S] cluster in CIAPIN1 is not dependent on GLRX3, regardless of their physical interaction [129].

The transient assembly of the Fe–S cluster on the Cfd1–Nbp35 scaffold is followed by the interaction of the heterotetrameric complex with the iron-only dehydrogenase Nar1, which receives the cluster to further pass it on to the targeting complex that will install the cluster into the apotargets [133]. Nar1 also bears an Fdx-like domain on its N-terminus and a cysteine domain on its C-terminus typical of iron-only hydrogenases (for binding of the H-cluster). Since Nar1 is itself an Fe–S cluster protein, its maturation depends on its association with the Cfd1–Nbp35 complex [133].

The following proteins constitute the CIA targeting complex in humans: the WD-40-type protein CIA1 (also called CIAO), which functions as a scaffold; MMS19 (Met18 in yeast), CIA2A (or FAM96A) and CIA2B (or FAM96B), whose role is targeting recipient apoproteins [15, 150–153]. The cytosolic iron–sulfur protein 1 (CIA1) belongs to the family of WD-40 proteins, which are known for their β-propeller structure repeats [150]. It was initially characterized in yeast, where its depletion affected the maturation of the cytosolic and nuclear Fe–S proteins, yet it had no impact on the levels of Nbp35. Moreover, Cia1 was found to interact with Nar1, the iron-only hydrogenase that receives the clusters from the Cfd1–Nbp35 scaffold. Similarly, ^55^Fe incorporation in the *Cia1* mutant affected apoproteins, such as Leu1 and Rli1, but had no significant impact on Nbp35, Nar1 and mitochondrial Fe–S proteins [150]. Complementation assays of the *Cia1* mutant with the human CIAO protein recovered the phenotype, confirming their functional redundancy. Moreover, a structural analysis of Cia1 determined that it exhibits a conserved propeller axis structure [154]. Hence, it was concluded that in the maturation of cytosolic Fe–S cluster proteins, Cia1 performs a role downstream of Nar1 [16].

MMS19 was initially discovered in yeast, in mutants with an increased sensitivity to methyl methasulfonate, as reflected by its name [155]. ^55^Fe incorporation in cells depleted for Mms19 affected apoproteins in a fashion similar to that described for the Cia1 mutant [151]. Importantly, the downregulation of MMS19 influenced proteins involved in the DNA metabolism, such as Rad3 helicase and XPD. In line with this finding, DNA helicase Dna2 and Rli1 were found interacting with MMS19, and the same applies to components of the CIA complex, namely CIA1, CIA2A and CIA2B [151, 152, 156]. Combined, these results provide evidence for the role of MMS19 downstream of CIA1, targeting a specific set of proteins, many of which are involved in DNA maintenance [151, 152].

Further characterization of proteins that were found to interact with MMS19 revealed their involvement in the late stages of the CIA pathway. Interestingly, CIA2A and CIA2B were found to interact in a mutually exclusive fashion with CIA1 [15]. Specific associations of these proteins seem to be necessary for the maturation of certain proteins. CIA2A is directly involved in the maturation of IRP1, which plays a role in cellular iron homeostasis [157]. CIA2B in association with CIA1 and MMS19 is responsible for the assembly of the Fe–S cluster of DYPD, an enzyme involved in the detoxification of pyrimidine derivatives [15]. Moreover, for the maturation of GPAT, an enzyme of the purine nucleotide synthesis pathway, and POLD, only CIA1 and CIA2B were required [15, 153].

MMS19 exhibits nine HEAT repeats, known to facilitate protein–protein interactions, which are distributed throughout the whole protein [153]. Mutations in the C-terminal HEAT repeats of MMS19 prove that they are responsible for the binding of CIA2B. Moreover, the absence of MMS19 renders CIA2B subject to proteosomal degradation [153]. Since the binding of CIA2B to MMS19 was often accompanied by the presence of CIA1, it was proposed that at least two distinct types of associations, namely CIA1–CIA2A and CIA1–MMS19–CIA2B, perform the maturation of different subsets of proteins (Fig. 1). In this model, CIA1 with CIA2A act in an iron-regulation/sensing role, as depicted by their specific interaction with IRP1 and IRP2, whereas CIA1 with MMS19 and CIA2B supply DNA metabolism-related proteins [15].

#### Iron sensing and regulation and the relationship with the CIA pathway

Iron sensing as a regulatory mechanism differs between mammals and yeast. In mammals, the iron-responsive protein 1 (IRP1) exhibits aconitase activity when bound to Fe–S clusters; once the cluster is lost, IRP binds to iron-responsive elements on mRNAs that specify proteins known to regulate iron homeostasis [157]. In yeast, aconitase does not exhibit mRNA binding activity. However, two important players related to the cytosolic Fe–S cluster assembly and iron homeostasis are the Grx3/4 proteins. They affect CIA proteins, their target apoproteins and some mitochondrial Fe–S assembly proteins, and therefore their involvement in cytosolic and mitochondrial iron homeostasis [158]. Yeast mutants Grx3/4 exhibit reduced activities of respiratory complexes II and IV, as well as those of the mitochondrial ISC assembly scaffold proteins Aco1, Leu1 and Isu1. Moreover, the mutants display up to tenfold decrease of iron incorporation into the CIA components that themselves carry Fe–S clusters, namely Dre2, Nar1 and their target protein Rli1, and the same applies for Isu1, biotin synthase, dihydroxyacid dehydratase and heme-containing catalase [158, 159]. These results point to an overall effect on iron supply, which was markedly more severe in the cytosol than in the mitochondria [158].

The regulation of iron concentration in yeast is directed by the transcription factors Aft1 and Aft2, which manage the transport of iron into the cell [160]. In iron-replete conditions, Aft1 is a cytosolic protein, yet when iron concentration is low, it is mobilized to the nucleus, where it activates the transcription of the iron regulon [160–162]. It has been suggested that this activation is incited by the failure of the mitochondrial iron supply directed toward the Fe–S cluster assembly, yet the deletion of components of the CIA pathway does not induce the same response [163, 164]. Mutants that activate the iron regulon by stimulating the activity of the high-affinity iron transport system of multicopper oxidase Fet3 led to the discovery of six proteins involved in this role [160, 165]. These are Nfs1, Isu1, Gsh2 [130], mitochondrial carrier Mtm1, an interactor of the multiprotein regulator complex (which in turns regulates RNA polymerase II), Sin4, [166] and Fra1 (Fe repressor of activation-1), the yeast homolog of bacterial BolA [165]. These findings confirm earlier observations, in which the deletion of Grx3 and Grx4 induced the iron regulon when iron concentrations were normal [161, 162]. Fra1 interacts with Fra2, Grx3 and Grx4, interactions that took place regardless of the iron conditions in which the cells were maintained [167]. It was also described that Grx3 and Grx4 interact with Aft1 [161]. Deletion of the gene *fra1* does not specifically activate the iron regulon, while that of *fra2* does [165]. Fra2 binds Grx3 through a specific interaction mediated via a histidine residue, which is critical for modulation of the iron regulon via Aft1 [168]. Based on this finding, Fra1 and Fra2 seem to interpret the iron conditions of the Fe–S cluster assembly. They are related to mitochondrial Fe–S cluster assembly and the iron regulon is induced when their concentration decreases [165].

In humans, only GLRX3 (also called PICOT) is found in the cytosol where it interacts with the cytosolic form of the BOLA family in humans, BOLA2 [129, 169]. This interaction is iron concentration dependent. Moreover, mutation of the GLRX3 cysteines in the motifs GrxA and GrxB, which are involved in binding of the [2Fe–2S] Fe–S cluster, almost completely disrupts the interaction with BOLA2 [129].

## Fe–S clusters in the supergroup Excavata

### Euglenozoa

Excavata is a eukaryotic “supergroup” that brings together a diverse array of protists [8, 170, 171], with just a few studied so far from the perspective of the Fe–S cluster assembly. The best-described one is that of the kinetoplastid flagellates, which are responsible for a range of diseases in humans and other vertebrates [172]. The only information available for the other two euglenozoan clades, the euglenids and diplonemids, can be derived from their genomes which are, however, not yet publicly available. All these protists display a single, usually extensively reticulated, cristae-bearing mitochondrion, with an active and complex electron transport chain. Kinetoplastids that belong to the genera *Trypanosoma* and *Leishmania* are capable of aerobic fermentation, as they excrete metabolites from both glucose and amino acid metabolisms, but use O_2_ as an electron acceptor [173]. Since both these parasitic groups exhibit a classical cytosolic Fe–S assembly, from the genomic information available to us we predict that similarly conserved situation occurs in diplonemids and euglenids (our unpubl. data).

**Fig. 2:**
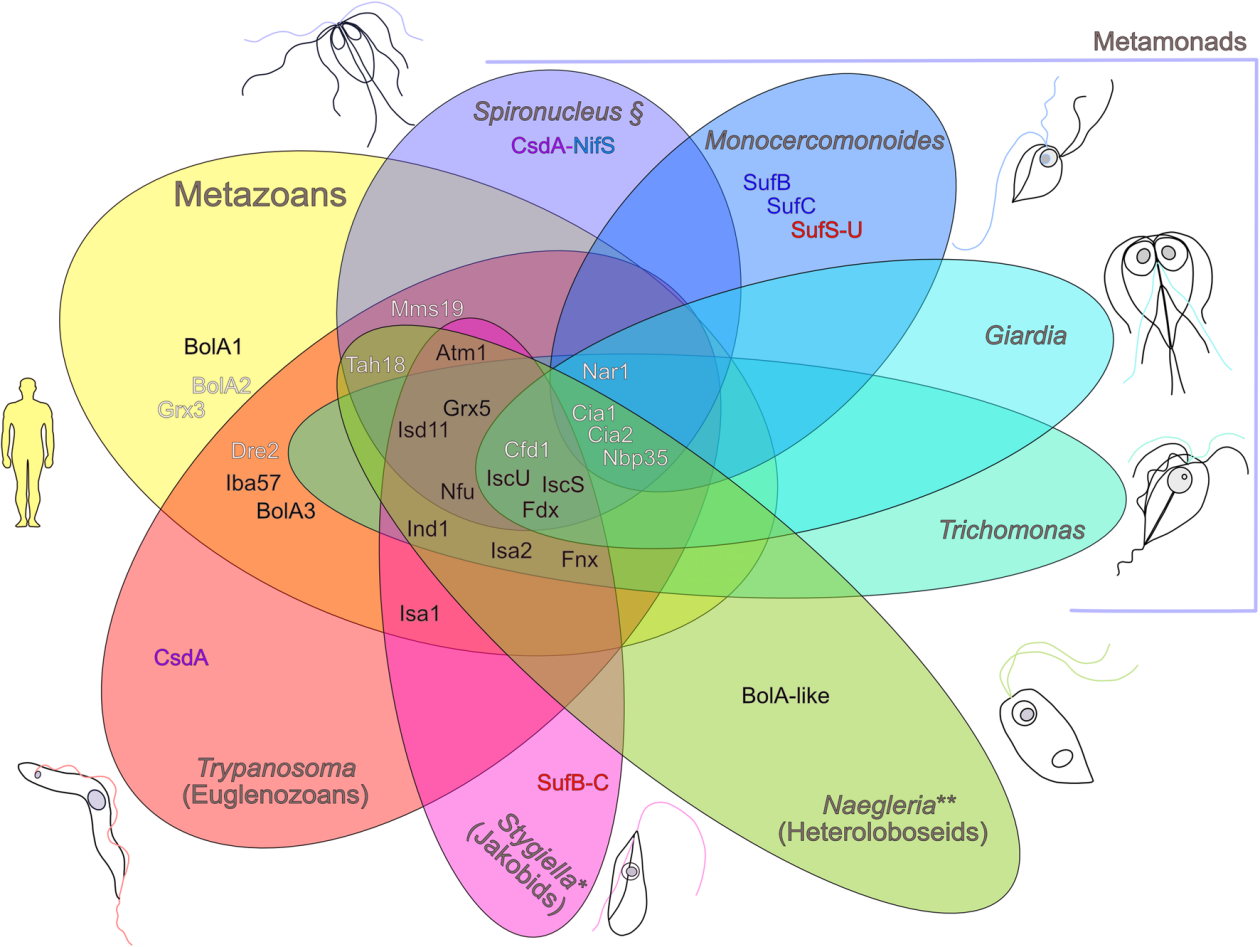
Distribution of ISC (black letters), CIA (white letters), NIF (light blue letters), CSD (purple letters) and SUF (in dark blue, gene fusions in red) components in metazoans and representative species of Excavata. Representative species denoted in the figure are Stygiella incarcerata*, Naegleria gruberi**, *Spironucleus salmonicida*§, *Monocercomonoides sp*., *Trichomonas vaginalis* and *Giardia intestinalis.* Data presented here should not be assumed for the whole genera, but only for representative species

The Fe–S cluster assembly was systematically studied only in the model species *Trypanosoma brucei,* which expresses all components of the ISC and CIA pathways in its mitochondrion and cytosol (Fig. 2) [172, 174]. Upon depletion, most of these proteins are essential for both studied life cycle stages, namely the bloodstream stage found in the blood of mammals and the procyclic stage that is confined to the tsetse fly vector. Moreover, rescues by human homologs or expression of the *T. brucei* proteins in the corresponding yeast mutants confirmed sequence-based homology predictions [20, 175–185], Tonini et al., resubmitted]. Only FdxB [177], selenocysteine lyase [78] and *Tb*Cia2A (Tonini et al. 2018, resubmitted) were found to be dispensable for *T. brucei.* A homolog of the Grx-interacting protein, BolA, has also been detected in the genome of *T. brucei*, although its role and localization have not been studied yet.

A number of gene duplications likely took place, as several components of this pathway are supernumerary. In particular, Nfu is represented by three paralogs, which are expressed in the mitochondrion and the cytosol in *T. brucei*, while four paralogs are found in the related *Leishmania* spp., although these remain unstudied [185]. Moreover, in *T. brucei* two ferredoxins (FdxA, FdxB) are present and transcribed [177], Mms19 is found in two genomic loci and Cia2 has been duplicated into Cia2A and Cia2B [172] [Tonini et al., resubmitted], just like in humans [15]. The presence of supernumerary components is not unique, since f.e. in the plant *Arabidopsis thaliana* five Nfu homologs are present in various subcellular compartments [119]. In some instances, the complexity took the path of alternatively spliced versions targeted at different locations, as was described in humans [118]. The finding that all three Nfu proteins are essential for *T. brucei* suggests that they are likely involved in the maturation of different sets of proteins [185].

Several aspects of the Fe–S cluster assembly and Fe–S cluster-carrying proteins have also been studied in the trypanosomatid parasite *Leishmania* spp., which unlike *T. brucei* prefers an intracellular niche that requires a range of specific metabolic adaptations [186]. *Leishmania* seems to use several Fe–S clusters containing proteins for redox sensing [187]. *Trypanosoma cruzi, T. brucei* and *Leishmania* spp. encode three 1-C-Grx genes in their genomes [188]. Monothiol (1-C-Grx) and dithiol (2-C-Grx) glutaredoxins exhibit distinct active sites motifs, CxxS and CxxC, respectively, which differentiate them [189]. It has been observed in vitro that in *T. brucei* three 1-C-Grxs proteins are capable of using glutathione and trypanothione as cofactors in the mitochondrial [2Fe–2S] cluster transfer [190]. This is a striking difference from the system described in yeast, where the glutaredoxin-bearing domain of Grx5 uses glutathione as a sulfur donor [188]. Even more remarkable is the fact that in *T. brucei* only 1-C-Grx1, but not 1-C-Grx2 and 3, is capable of partially rescuing the deleterious phenotype of the Grx5 yeast mutant [188]. All other components of the ISC pathway, as well as most proteins known to participate in the CIA pathway, have also been detected in the *Leishmania* genome [74].

### Heteroloboseids

This clade brings together amoebic, amoeboflagellated and cyst-forming protists found in both oxic and anoxic (or microaerophilic) environments, most of which remain understudied [191]. Approximately, 150 species are known to belong to this clade and, though it is not species rich, this group gathers a high morphological variability and a broad ecological distribution. Some of its members may transit through the flagellated and amoeba life stages, while others have retained either the former or the latter stage [191]. They have been found in a wide range of environmental conditions such as extreme heat, cold, high altitudes, extreme pH and hypersaline concentrations [192–197]. We know most about the genus *Naegleria*, as it includes the brain-infecting amoeba *N. fowlerii,* and the related *Paravahlkampfia francinae,* also responsible for amoebic meningoencephalitis [198]. Both species possess classical mitochondria. On the other hand, a group of heteloboseids, such as *Sawyeria marylandensis* [199, 200], *Psalteriomonas lanterna* [201–203]*, Monopylocystis visvesvarai* [199] and *Creneis carolina* [204], are found mostly in low oxygen conditions and carry the MROs. Other heteroloboseids including the halophilic *Pleurostomum flabellatum* and *Vahlkampfia anaerobica* have been described to harbor cristae-devoid mitochondria, although their genomes and metabolisms have not yet been thoroughly studied [193, 205].

*Naegleria* possesses classical ATP-producing mitochondria and displays components of the ISC pathway IscS, IscU and Isd11, as well as two putative Fdxs, Isa, Grx, Atm1 and a BolA-like protein (Fig. 2). Moreover, the hydrogenosome of *Sawyeria marylandensis* contains paralogs of IscS and IscU, as well as Fxn and Fdx [200].

### Jakobids

Mitochondrial genomes of jakobids belong to the most bacterial-like genomes known to date, though this premise has been challenged by the plausibility of extensive lateral gene transfer into the mitochondrial genomes [206–208]. Regardless, the organelle of jakobids contains the most gene-rich mitochondrial genomes, with the most extreme case represented by *Andalucia godoyi,* which displays 66 protein-coding genes and 29 tRNA genes [209]. The organelle of *Stygiella incarcerata,* originally considered to be a mitochondrion devoid of the cristae, is now known to be a DNA-lacking hydrogenosome [207, 210].

Interestingly, the hydrogenosome of *S. incarcerata* retains a complete Fe–S cluster assembly machinery, which includes IscS, IscU, Isa1 and Isa2, Fxn, Fdx, Grx5, Ssq1 chaperone and Nfu (Fig. 2, Table 1) [210]. The ABC transporter Atm1 is also expressed, suggesting this organism displays a mitochondrion-dependent, cytosol-localized Fe–S cluster assembly. At the same time, a SufCB fusion seems to lack the mitochondrial targeting signal, implying its highly likely cytosolic localization [210]. However, it has been documented in other MRO-bearing organisms such as *Blastocystis, Entamoeba* and *Trichomonas* that the hydrogenosomal proteins may not require a dedicated targeting signal to be translocated into the organelle [28, 211–213]. The assembly of SufBCD in bacteria studied both in vitro and in vivo demonstrated its role in the maturation of [2Fe–2S] Fdx, aided by SufA in the transfer of the cluster [48–50]. The SufCB fusion, most likely obtained from bacteria, has been documented only in the cytosol of the anaerobic pathogen *Blastocystis* [22], in the MRO lumen of *Pygsuia biforma* [13] and *Stygiella* [210]. The other bacterial machinery, the NIF pathway, has been observed replacing the ISC system of *Entamoeba histolytica* [77] and *Mastigamoeba balamuthi* [76].

### Metamonada

This group consists of anaerobic or microaerophilic protists and is formed by three main lineages: (1) Fornicata (or the diplomonad/retortemonad/carpediemonad clade), represented in this review by the mitosome-bearing *Giardia intestinalis* and *Spironucleus salmonicida* [214]; (2) Parabasalia, with the flag species *Trichomonas vaginalis*, bearer of the first-described hydrogenosome [6]; (3) Preaxostyla, denoted by *Monocercomonoides* [23] and the free-living *Paratrimastix* [215]. In all of Excavata, Metamonada exhibits the greatest functional diversity of MROs [28].

*Giardia* mitosomes lack DNA and are unable to generate or export ATP into the cytosol [5]. Nevertheless, they possess classic ISC components, such as IscS, IscU, Isa, Nfu and Grx [216–221]. Although incomplete, most of the CIA pathway of *Giardia* is localized in the cytosol, with three homologs of Nbp35 (Nbp35-1, Nbp35-2 and Nbp35-3), while Dre2, Tah18 and Mms19 seem to be absent (Table 1) [143]. Moreover, the ortholog of Cia2 exhibits dual localization in the intermembrane space of the mitosome and the cytosol, while Npb35 seems to be associated with the cytosolic side of the outer mitosomal membrane [143]. It was rather unexpected to find the CIA pathway in a cell lacking Atm1 and its cytosolic interactor, the Tah18/Dre2 complex, as it is widely accepted that the CIA pathway depends on a sulfur-containing molecule transported from the mitochondrial matrix into the cytosol via Atm1. Therefore, it has been hypothesized that the dual localization of Cia2 aids in the transport of the sulfur-containing molecule necessary for the cytosolic assembly, in the apparent absence of the ABC transporter Atm1 [143]. The conspicuous absence of these proteins has also been observed in flagellates where the SUF or the NIF system has replaced the ISC pathway, such as *Pygsuia* and *Blastocystis* [13, 22].

*Spironucleus salmonicida* encodes an unusually high number of cysteine-rich proteins and evolved an extended system for counteracting oxygen stress [214]. This diplomonad exhibits a standard hydrogenosome with a classical MRO-type ISC pathway [222, 223]. All of its components, namely IscS, IscU, Nfu, Fxn, two Fdxs and Jac1, have been localized to the organelle and bear MRO targeting signals [222]. Moreover, a selenocysteine lyase has been detected in this parasite, although neither its expression nor its localization has been confirmed; still, a sequence of a fused selenophosphate synthetase–NifS is present in its genome, similar to the one found in *T. brucei* [78, 214]. Putative components of the CIA pathway are also found in the *S. salmonicida* genome, such as Cfd1, Nbp35, Tah18, Nar1, Cia1, Mms19 and Cia2 (Fig. 2, Table 1).

The human parasite *T. vaginalis* is a microaerophile, which exhibits a very strong iron-regulatable gene expression that allows it to modulate virulence factors and its ability to adapt to microenvironments upon infection [224–227]. Changes in the iron concentrations of its environment result in fold changes in the expression of Fe–S clusters assembly components [224, 225]. The DNA-lacking hydrogenosome of *T. vaginalis* [228] harbors the ISC-type Fe–S cluster assembly and makes use of a supernumerary machinery of components: IscU, two IscS, Isd11s and Fxns, seven [2Fe–2S] Fdxs, three Isa2, four Nfus, four Ind1s (Table 1), as well as two homologs of the chaperone Hsc20 [24, 225, 229–231]. Upon iron depletion, most of these components become upregulated at least twofold. An example of this situation is Fdx, which in this flagellate exhibits six orthologs, of which half is upregulated and half is down-regulated during low iron conditions. This led to the assumption that the expression levels would relate them to their putatively different function, namely a role in the Fe–S cluster assembly of the upregulated members, whereas those with low expression are likely involved in metabolic activities [225].

*Trichomonas vaginalis* is the only member of Excavata and the only other eukaryote apart from *Entamoeba histolytica* to express a homolog of the bacterial-type iron–sulfur flavoprotein (Isf) [232]. This hydrogenosomal protein is involved in ROS detoxification and protection from the oxygen-rich environmental changes. Moreover, it seems to be present only in methane-synthesizing archaea and bacteria [233, 234]. Isf is able to reduce oxygen and has a detoxifying activity against the drugs metronidazole and chloramphenicol, used to treat anaerobic infections like those caused by *T. vaginalis* [232]. Isf is able to receive in vitro electrons from Fdx and NADH [232]. Like other metamonads, *T. vaginalis* also lacks the Tah18/Dre2 complex, along with Mms19, while it expresses orthologs of Cfd1, Nbp35, Nar1, Cia1 and Cia2 (Table 1) [143].

Various other metamonads, namely *Carpediemonas membranifera, Chilomastix cuspidata, Dysnectes brevis, Ergobibamus cyprinoides, Spironucleus vortens* and *Trepomonas PC1,* for which no complete genomes but expressed sequence tags are available, bear IscS and IscU [28]. Moreover, they all seem to express Nbp35 of the CIA pathway, while Cfd1 is mostly absent in this group, with the exception of *C. membranifera* and *C. cuspidata* [143]. Cia1 was found in all these protists, except *S. vortens*. Like in a range of metamonads, the Tah18/Dre2 complex and Mms19 are absent. However, it has been suggested that the remaining CIA components may be present in the genomes, but missed in the available datasets [143].

Finally, the oxymonad *Monocercomonoides,* the only genuine “amitochondriate” known so far, is also the most extreme example of the Fe–S cluster machinery reduction [23]. With several CIA pathway components complemented by the expression of SufB, SufC and the SufS–U fusion, it is the only known eukaryote that completely lacks the mitochondrial ISC machinery and instead acquired the bacterial-type SUF components. None of these components seem to bear a MRO targeting signal, as they translocate neither into the hydrogenosome of *T. vaginalis* nor the mitochondria of *S. cerevisiae* [23]. The CIA pathway components of *Monocercomonoides* identified so far are Nbp35, Nar1, Cia1 and Cia2A and Cia2B (Table 1). Its composition implies that the de novo formation of clusters uses the bacterial SufS–U CD-scaffold on SufB and SufC, and was apparently obtained by a lateral gene transfer. This event likely took place before the loss of the mitochondrion in *Monocercomonoides*, as gathered from its closest relative, *Paratrimastix pyriformis*, which also possesses homologs of the SUF machinery and bears an MRO [23, 235].

## Presence vs functionality: the requirement for mechanistic data to complement phylogenomic analyses

The expansion of genomic analysis tools has given rise to a high number of publicly available genomes. Studies of the evolution of mitochondria and MROs have unraveled a wide variety of adaptations in the Fe–S cluster assembly machineries that could provide explanations for the substantial differences observed even among the model organisms from the supergroup Opisthokonta. The mechanisms behind the presence or absence of components of the ISC, SUF and CIA pathway in these clades, however, are mostly unknown, basically due to lack of mechanistic data.

**Table 1:** Presence/absence of ISC, CIA, SUF, NIF and CSD components in different excavates and breviate *Pygsuia*

	Organism	*T. brucei*	*L.donovani*	*Naegleria*	*Sawyeria* ^a^	*Stygiella* ^a^	*Giardia* ^a^	*Spironucleus* ^a^	*Trichomonas* ^a^	*Monocercomonoides* ^a^	*Pygsuia*
ISC	IscS	✓	✓	✓	✓	✓	✓	✓	✓		✓
	IscU	✓	✓	✓	✓	✓	✓	✓	✓		
	Isd11	✓	✓	✓		✓		✓	✓		
	Isa1	✓				✓					
	Isa2	✓				✓			✓		
	Fdx	✓	✓	✓	✓	✓	✓	✓	✓		
	Fnx	✓	✓	✓	✓	✓		✓	✓		
	Nfu	✓	✓			✓	✓	✓	✓		✓
	Iba57	✓									
	Ind1					✓			✓		✓
	Atm1	✓	✓	✓		✓		✓			
CIA	Cfd1	✓	✓	✓	?	✓		?	✓		✓
	Nbp35	✓	✓	✓	?	?	✓	✓	✓	✓	✓
	Dre2	✓	✓	✓	?	?		?			
	Tah18	✓	✓	✓	?	?		?			
	Nar1	✓	✓		?	?	✓	✓	?	✓	✓
	Cia1	✓	✓	✓	?	?	✓	✓	✓	✓	✓
	Cia2	✓	✓	✓	?	?	✓	✓	✓	✓	✓
	Mms19	✓	✓		?	?		?			✓
SUF	SufS									✓	
	SufU										
	SufB					✓				✓	✓
	SufC									✓	
NIF	NifS							✓			
CSD	CsdA	✓									

^a^The data corresponds to representative species (should not be assumed for the whole genera): *Sawyeria marylandensis, Stygiella incarcerata, Giardia intestinalis, Spironucleus salmonicida, Trichomonas vaginalis, Monocercomonoides* sp.

✓ indicates presence; ? denotes the gene/protein has not been found, but may be present; blank indicates absence

One of the prevalent differences observed between the few above-mentioned species and the Opisthokonta model species is the absence of Isd11 in organisms bearing ISC. This feature points out to an acquisition of IscS/IscU from bacterial lineages. Regardless, in Excavata IscS is always accompanied by IscU, which confirms the findings of several groups on the requirement of the CD for its specific scaffold to perform desulfuration of cysteine [236]. The presence of Isd11 in *T. vaginalis* has been suggested as a unique acquisition, from a endosymbiotic event that gave origin to MROs [79]. However, this hypothesis leaves out the fact that the protein is also absent in protists described recently as bearing similar organelles.

The dispersed distribution of the scaffold-type proteins, particularly those characterized as the carrier or transfer proteins, is important for the determination of the mechanistic specificity of the Fe–S cluster assembly systems. CsdE is a CD incapable of complementing the assembly of Fe–S clusters on the IscU scaffold, despite the wide range of substrates this CD is able to metabolize [46, 71, 73, 236]. This is, however, not exactly the same for the transfer proteins, which to deliver the clusters also require a physical interaction with their recipient apoproteins. Unlike the CDs with U-type scaffolds, the carrier or transfer proteins seem to be, to a certain extent, interchangeable in bacteria [73]. For example, the deletion of IscA or SufA in *E. coli* does not affect survival; however, this is not true for the absence of ErpA [237, 238]. A similar situation was observed for *S. cerevisiae* [239]. However, in the presence of oxygen, *Azotobacter vinelandii* cannot survive mutation of IscA in the presence of oxygen [240]. The distinct phenotypes following the deletions or mutations of carrier proteins in bacteria indicate their functional specificity in vivo [53, 237]. This means that the recipient proteins that require maturation by specific transfer proteins may or may not be structurally recognized by a given system component [53]. These cellular requirements cannot be bypassed by the apparent overall functional redundancy of the Fe–S assembly systems. The case of *Pygsuia* is a notable exception to these observations. Breviate *Pygsuia* exhibits a SufB–C fusion, yet it also harbors a copy of IscS, with no obvious presence of IscU. Therefore, this fusion scaffold may be capable of providing the structural functionality for IscS. [13].

A scaffold protein that has been conserved throughout evolution is Nbp35. Although the CIA pathway displays the characteristics of a relatively new eukaryotic acquisition, the almost universal presence of this protein conveys how the Fe–S cluster assembly can repurpose a pre-existing protein for a new function. On the other hand, the partner of Nbp35, Cfd1, has a rather patchy distribution in the protist groups. Originally from Archaea, most ApbC homologs in bacteria and archaea lack the Fdx motif present in Nbp35 [139].

Another interesting finding is the gene fusion of various SUF components present in metamonads and jakobids. The SUF system is the most ancient of all Fe–S cluster assembly systems [9]. Its wide distribution among the aerobic and anaerobic organisms led to the proposal that it evolved prior to oxygenation of the biosphere and adopted mechanisms for counteracting oxidizing conditions [241]. Though the SUF and ISC systems coexist in various bacterial lineages, the complementation with SUF components in the absence of specific ISC components has not been possible [242]. It has been well established that in proteobacteria the Fe–S cluster assembly by the SUF pathway is highly regulated by protein–protein interactions [55, 236, 243]. A study that reported the complementation of the SUF system of *E. coli* with that of *E. faecalis* also conveyed that the SUF machinery of the Gram-positive bacteria could replace IscS neither in *E. coli* nor in *A. vinelandii* [242]. These features pinpoint to a specificity of activities, which may explain why in the protist systems with the SUF fusions IscS or IscU are absent. The presence of the SUF systems in eukaryotes is documented in plastids, which also lack ISC [244]. However, this system displays functional differences from that of bacteria. The observation that SufB in *A. thaliana* has an ATPase activity, unlike its counterpart in bacteria, denotes that in eukaryotes the SUF system has acquired unique features [245, 246].

The hypothesis mentioned above gives a mechanistic answer for the characteristic presence of the SUF system in protists. However, there is no explanation as to why the SUF system, rather than the ISC or the NIF systems, was obtained. It has been reported that the relationship between the SUF and ISC systems involves regulation of the complete pathways based on environmental conditions. Such is the case of the SUF operon in *E. coli*, which is overexpressed on low-iron conditions and oxidative stress, at the same time that the ISC operon is rendered inactive by transcriptional regulation of IscR. In *Pseudomonas aeruginosa*, IscR regulates cellular iron homeostasis by sensing Fe–S concentrations [247]. *T. vaginalis* is an example of adaptation of the Fe–S cluster machinery to match specific cellular requirements. This anaerobic parasite displays supernumerary components of various components of the ISC pathway, which are up- or down-regulated in response to the changing iron concentrations in its environment [225].

Therefore, to characterize the regulatory capacity of a system, one has to determine the expression of its components. However, this type of data may be obtained only by analyzing the Fe–S cluster assembly as a whole, a field where microbiologists take the lead when compared with what is known about eukaryotic systems. This should not be confused with analytical methods, which are rich in studies of the Fe–S cluster assembly in Opisthokonta. Still, the limited set of model organisms restricts our overall picture. While it is challenging to establish the methodology for a new eukaryotic model organism, this has to be overcome. The establishment of evolutionary relationships based on proven experimental models nicely complements the increasing available genomic data, providing elegant mechanistic explanations for changing phylogenomic models [248, 249].

## Closing remarks

The reduction of mitochondria has seen the relocation of the Fe–S cluster assembly to about elsewhere in the cell or a general replacement of its components with paralogs from diverse proteobacterial lineages. The notion that the ISC has been lost from MROs does not translate into the same fate for the mechanism in question, in this case the Fe–S cluster assembly. It translates into an adaptation to maintain the entire mechanism, more than to strictly conserve a given set of genes of certain origin. It will require functional analysis of the pathway in some of the protists mentioned above to define the flexibility and limits of simplification and/or patching of the Fe–S cluster assembly, which from all we know is an indispensable component of all extant life.
