# Correction to: Fe–S cluster assembly in the supergroup Excavata

**DOI:** 10.1007/s00775-018-1568-2

**Published:** 2018-05-29

**Authors:** Priscila Peña-Diaz, Julius Lukeš

**Affiliations:** 1 Institute of Parasitology, Biology Centre, Czech Academy of Sciences, České Budějovice (Budweis), Czech Republic; 2 Faculty of Sciences, University of South Bohemia, České Budějovice (Budweis), Czech Republic

## Correction to: JBIC Journal of Biological Inorganic Chemistry 10.1007/s00775-018-1556-6

The article “Fe–S cluster assembly in the supergroup Excavata”, written by Priscila Peña‑Diaz, Julius Lukeš was originally published electronically on the publisher’s internet portal (currently SpringerLink) without open access.

The copyright of the article changed on 25, May to © The Author(s) 2018 and the article is forthwith distributed under the terms of the Creative Commons Attribution 4.0 International License (http://creativecommons.org/licenses/by/4.0/), which permits use, duplication, adaptation, distribution and reproduction in any medium or format, as long as you give appropriate credit to the original author(s) and the source, provide a link to the Creative Commons license and indicate if changes were made.

The original article has been corrected.

